# Supplementary material for: Structural and mechanistic insights into regulation of the retromer coat by TBC1d5
Source: Nat Commun. 2016 Nov 9;7:13305. doi: 10.1038/ncomms13305 (PMC5105194; doi:10.1038/ncomms13305)
Supplement: Supplementary Information — Supplementary Figures 1-10, Supplementary Tables 1-3 and Supplementary References. [file ncomms13305-s1.pdf]

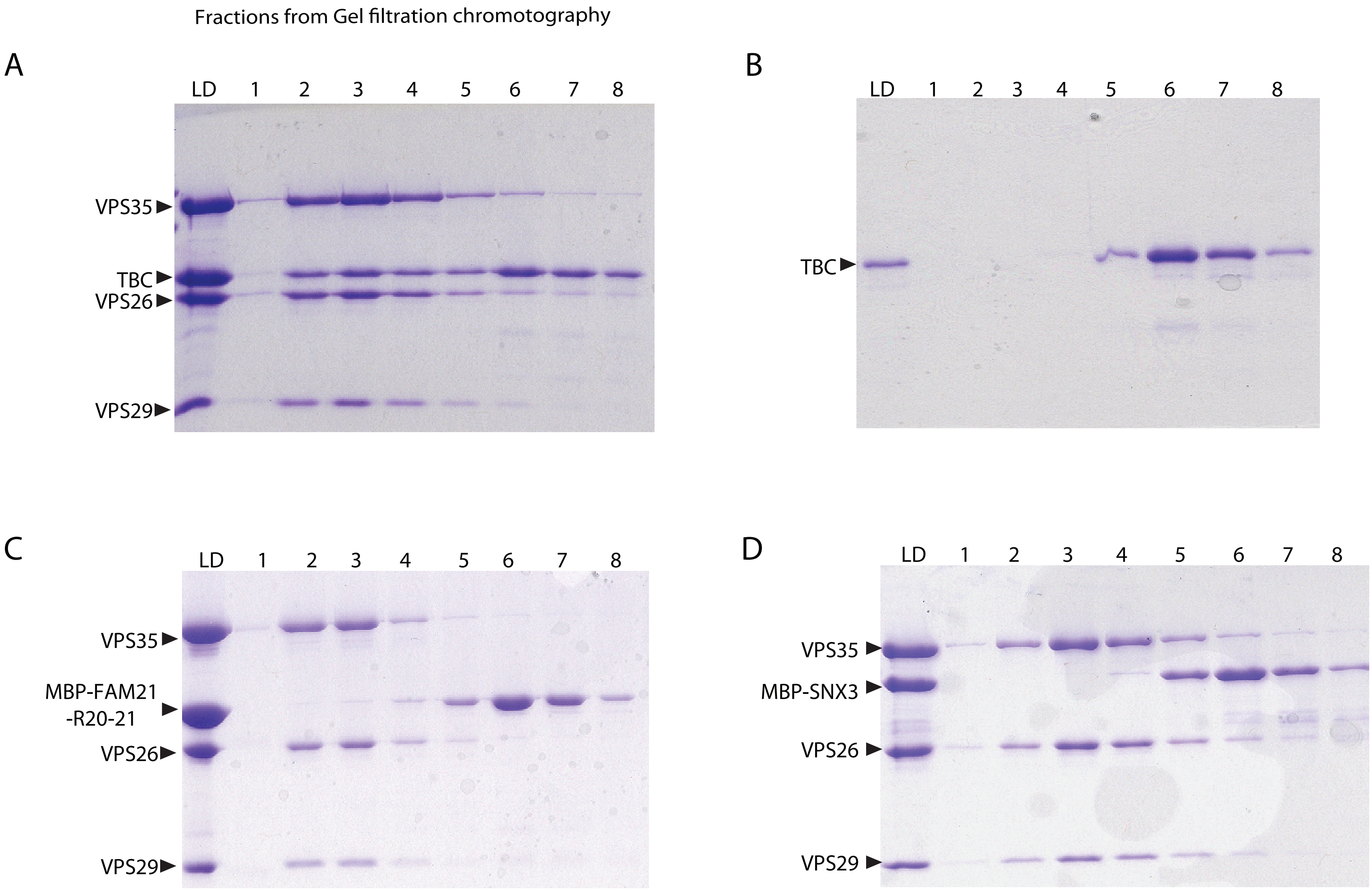
Fractions from Gel filtration chromotography
A
LD 1 2 3 4 5 6 7 8
VPS35
TBC
VPS26
VPS29
B
LD 1 2 3 4 5 6 7 8
TBC
C
LD 1 2 3 4 5 6 7 8
VPS35
MBP-FAM21
-R20-21
VPS26
VPS29
D
LD 1 2 3 4 5 6 7 8
VPS35
MBP-SNX3
VPS26
VPS29

Supplementary Figure 1. TBC1d5, but not FAM21 and SNX3, forms a stable complex with CSC.

Gel filtration chromatography of samples containing purified CSC and TBC1d5^{TBC} (A), or MBP-FAM21-R20-21 (C), or MBP-SNX3 (D) at a molar ratio of 1:2, or TBC1d5^{TBC} by itself (D). Commassie blue stained SDS-PAGE gels are shown for the corresponding fractions from a 24-mL Superdex200 column. The column was euilibrated and eluted in GF buffer (20 mM Tris-HCl, pH 8.0, 200 mM NaCl, 5 mM βME). R20-21 are the tighest CSC-binding motif among 21 repeats of FAM21, and MBP fusions were used to lift the size of FAM21 and SNX3.

```
TBC1D5        MYHSLSETRHPLQPEEQ-EVGIDPLSSYSNKSGGDSNKNGRRT--------SSTLDSEGT     51
TBC1D22A      --ELLAMAAESLNSEVVMETANRVLRNHSQRQGRPTLQEGPGLQQKPRPEAEPPSPPSGD     118
TBC1D13       --------------------------------------------------MSSLH----     5

TBC1D5        FNSYRKEWEELFV-NNNYLATIRQKGINGQLRSSRFRSICWKLFLCVLPQDKSQWISRIE     110
TBC1D22A      LRLV-KSV--SESHTSCPAEELRRLSWSGIPK--PVRPMTWKLLSGYLPANVDRRPATLQ     173
TBC1D13       -KSRIADFQDVLKEPSIALEKLRELSFSGIPCEGGLRCLCWKILLNYLPLERASWTSILA     64
                                    *     *       *   **     **

TBC1D5        ELRAWYSNIKEIHITNPRKV------VGQQDLMINNPLSQDEGSLWNKFFQDKELRSMIE     164
TBC1D22A      RKQKEYFAFIEHYY------------------------DSRNDE----VH--QDTYRQIH     143
TBC1D13       KQRELYAQFLREMIIQPGIAKANMGVSREDVTFEDHPLNPNPDSRWNTYFKDNEVLLQID     124
                   *                                                    *

TBC1D5        QDVKRTFPEMQFFQQENVRK----------------------------------------     184
TBC1D22A      IDIPRMSPEALILQP---------------------------------------------     218
TBC1D13       KDVRRLCPDISFFQRATDYPCLLILDPQNEFETLRKRVEQTTLKSQTVARNRSGVTNMSS     184
               *  *  *     *

TBC1D5        -------------------------ILTDVLFCYARENEQLLYKQGMHELLAPIVFVLHC     219
TBC1D22A      ---------------------KVTEIFERILFIWAIRHPASGYVQGINDLVTPFFVVFIC     257
TBC1D13       PHKNSVPSSLNEYEVLPNGCEAHWEVVERILFIYAKLNPGIAYVQGMNEIVGPLYYTFAT     244
                                            **  *       * **      *

TBC1D5        DHQAFLHASESAQPSEEMKTVLNPEYLEHDAYAVFSQLMETAEPWFSTFEHDGQKGKETL     279
TBC1D22A      EYIEAEEVDTVDVSGV---PAEVLCNIEADTYWCMSKLLDGIQDNYTFAQPGIQMK----     310
TBC1D13       DPNSEWK-----------------EHAEADTFFCFTNLMAEIRDNFIKSLDDSQCG----     283
                                         * *       *               *

TBC1D5        MTPIPFARPQDLGPTIAIVTKVNQIQDHLLKKHDIELYMHLNRLEIAPQIYGLRWVRLLF     339
TBC1D22A      --------------------VK---MLEELVSRIDEQVHRHLDQHEVRYLQFAFRWMNNLL     348
TBC1D13       -----------------ITYKME-KVYSTLKDKDVELYLKLQEQNIKPQFFAFRWLTLLL     325
                                  *            *      *            **   *

TBC1D5        GREFPLQDLLVVWDALFADGLSLG-LVDYIFVAMLLYIRDALI-SSNYQTCLGLLMHYPF     397
TBC1D22A      MREVPLRCTIRLWDTYQSEPDGFSHFHLYVCAAFLVRWRKEILEEKDFQELLLFLQNLPT     408
TBC1D13       SQEFLLPDVIRIWDSLFADDNRFD-FLLLVCCAMLMLIREQLL-EGDFTVNMRLLQDYPI     383
                *  *      **                  * *   *               *   *

TBC1D5        IGDV---HSL-ILKALFL-----RDPKRNPR        419
TBC1D22A      AHWDDEDISLLLAEAYRLKFAFADAPNHYKK        439
TBC1D13       TDV----CQI-LQKAKEL-----QDSK----        400
```

Supplementary Figure 2. Sequence comparision of TBC1d5, TBC1d22A, and TBC1d13. TBC1d22A and TBC1d13 are the two closest homologs to TBC1d5 among over 40 human TBC-containing proteins. Sequence alignments were performed with ClustalW, with * for invariant amino acids and Ins1 and Ins2 highlighted in red.

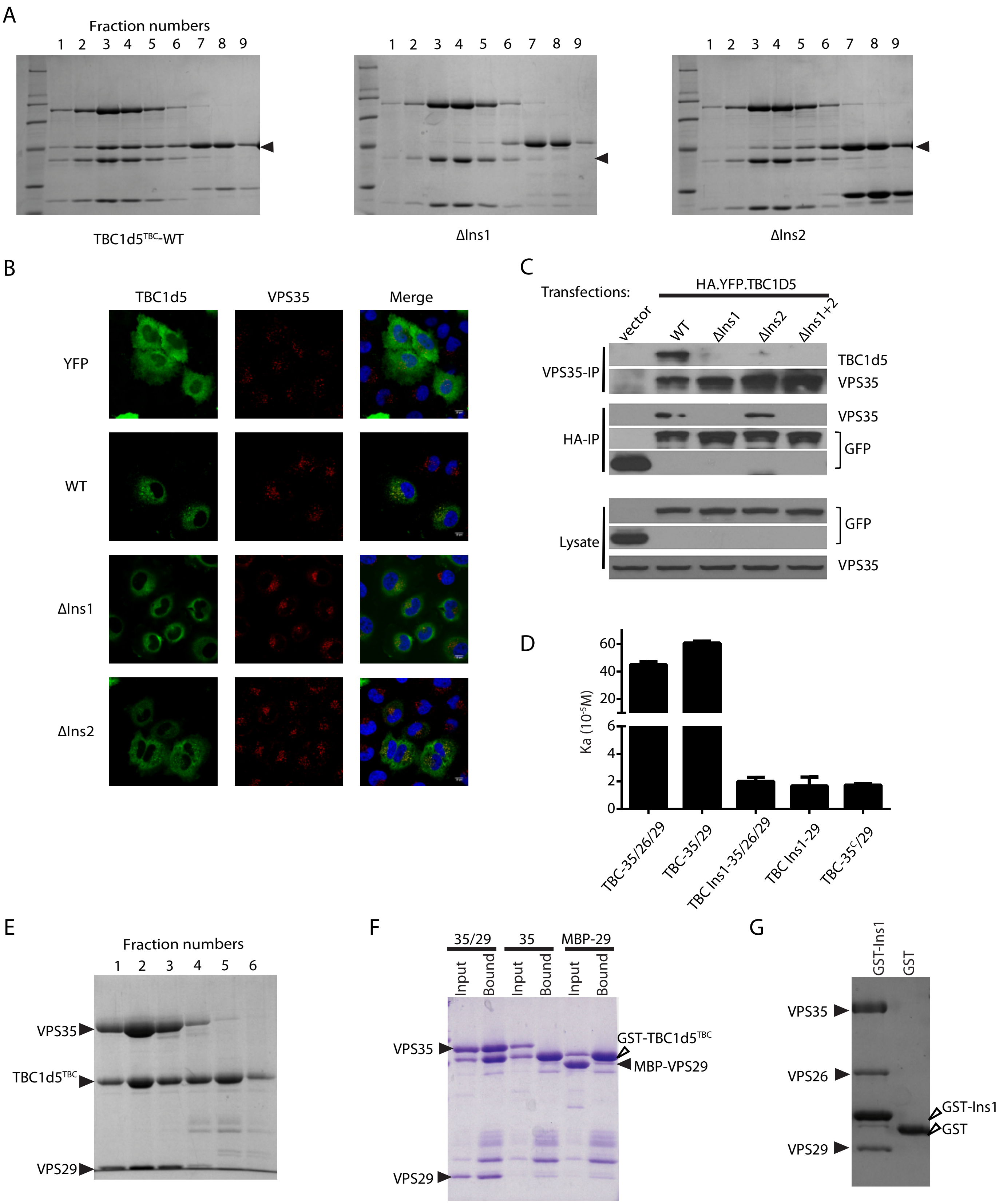

A
Fraction numbers
1 2 3 4 5 6 7 8 9
TBC1d5TBC-WT
ΔIns1
ΔIns2
B
TBC1d5
VPS35
Merge
YFP
WT
ΔIns1
ΔIns2
C
Transfections:
HA.YFP.TBC1D5
vector
WT
ΔIns1
ΔIns2
ΔIns1+2
VPS35-IP
HA-IP
Lysate
TBC1d5
VPS35
GFP
D
Ka (10-5M)
60
40
20
6
4
2
0
TBC-35/26/29
TBC-35/29
TBC Ins1-35/26/29
TBC Ins1-29
TBC-35C/29
E
Fraction numbers
1 2 3 4 5 6
VPS35
TBC1d5TBC
VPS29
F
35/29
35
MBP-29
Input
Bound
GST-TBC1d5TBC
MBP-VPS29
G
GST-Ins1
GST
VPS26

Supplementary Figure 3. Map the interaction between CSC and TBC1d5**.**

(A) TBC1d5^{TBC} wild-type, but not ΔIns1 and ΔIns2, co-migrated with CSC on gel filtration chromatography. Gel filtration chromatography of samples containing CSC and TBC1d5^{TBC} at a molar ratio of 1:2. Commassie blue stained SDS-PAGE gels are shown for the corresponding fractions from a 24-mL Superdex200 column. Sale bar=10 μm.

(B) Subellular localization of TBC1d5 wild-type, ΔIns1, and ΔIns2. Hela cells were transfected with YFP, or various YFP-TBC1d5 (green), and then fixed and labeled with anti-VPS35 antibodies (red).

(C) TBC1d5^{TBC} wild type, but not ΔIns1, ΔIns2, and ΔIns1+2, co-immunoprecipitated with VPS35. Hela cells were transfected with YFP, or various HA-YFP-TBC1d5 proteins, and immunoprecipitated with anti-VPS35 or anti-HA antibodies. Lysates were immunoblotted as a control, and anti-GFP antibodies were used to detect YFP.

(D) Affinity between various CSC sub-complexes and TBC1d5^{TBC} (TBC) or Ins1 determined by Isothermal titration calorimetry. Association constant (Ka) are shown together with errors from data fitting.

(E) TBC1d5^{TBC} co-migrated with VPS35/VPS29 on gel filtration chromatography. Gel filtration chromatography of samples containing VPS35/VPS29 and TBC1d5^{TBC} at a molar ratio of 1:2. Commassie blue stained SDS-PAGE gels are shown for the corresponding fractions from a 24-mL Superdex200 column.

(F) Commassie blue stained SDS-PAGE gels show that GST-TBC1d5^{TBC} preferentially retained VPS35/VPS29 over VPS29 or VPS35.

(G) Commassie blue stained SDS-PAGE gels show that GST-TBC1d5-Ins1 selectively retained CSC. Commassie blue stained SDS-PAGE gels for the bound samples are shown.

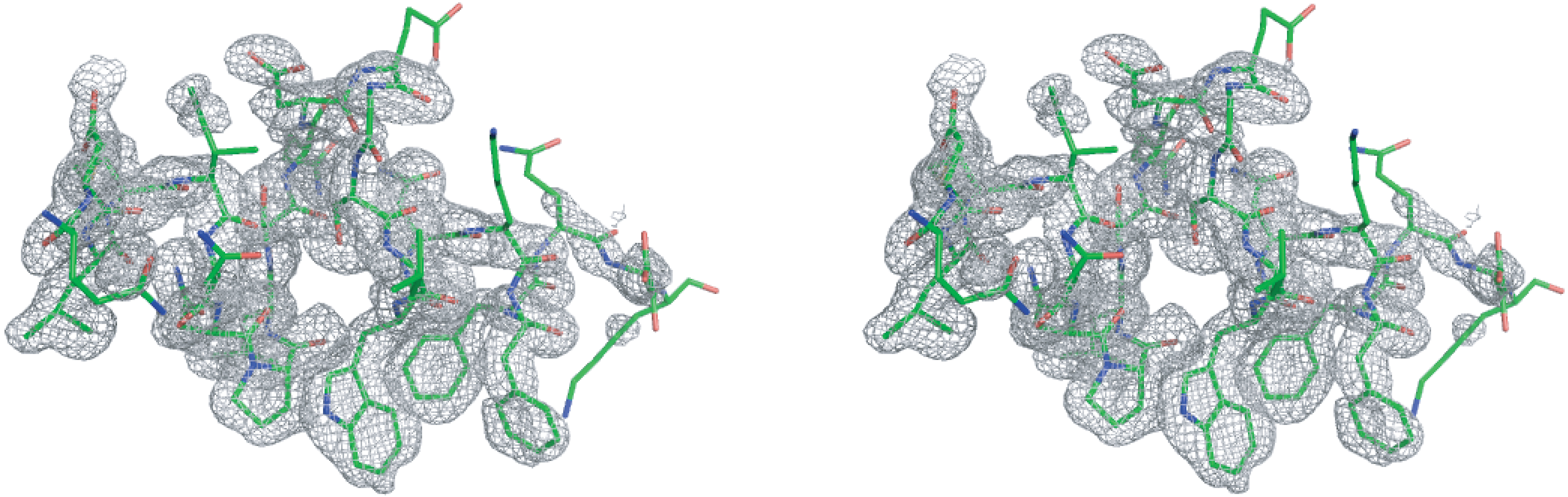

Supplementary Figure 4. Stereo view of overlay between TBC1d5-Ins1 peptide with a composite Simulated Annealing omit map at 1.5 Å resolution and contoured at 1σ.

A

```
H.sapiens        132 GQQDLMINNPLSQDEGSLWNKFFQDKE 158
M.musculus       132 GQQDLMINNPLSQDEGSLWNKFFQDKE 158
D.rerio          124 GQQDLVVNNPLSQDEGSLWNKFFQDKE 150
D.melanogaster   119 QLAVDCNDDPLSQSTQSVWNQYFSDQE 145
A.thaliana        72 NSPDLSIDNPLSQNPDSTWGRFFRNAE  98
C.elegans         91 FSQDPEFNNPLASIEQNPWNTFFEDND 117
E.histolytica     94 TEESDTIPDPLSINENNPWCQHFNEMD 120
S.Pombe           88 EESGENSDHPLNTSDDSKWKEYFDDNQ 114
                             .**     . *  .* : :
```

B

```
H.sapiens            ----MLVLVLGDLHIPHRCNSLPAKFKKLL-VPGKIQHILCTGNLCTKESYDYLK---TL 52
M.musculus           ----MLVLVLGDLHIPHRCNSLPAKFKKLL-VPGKIQHILCTGNLCTKESYDYLK---TL 52
D.rerio              ----MLVLVLGDLHIPHRCNTLPAKFKKLL-VPGKIQHILCTGNLCTKESYDYLK---TL 52
D.melanogaster       ----MLVLVLGDLHIPHRCSSLPAKFKKLL-VPGRIHHILATGNICTKESYDYLK---SL 52
A.thaliana           ---MVLVLALGDLHVPHRAADLPPKFKSML-VPGKIQHIICTGNLCIKEIHDYLK---TI 53
C.elegans            MLNFQLVLLIGDFNLPHRAANISPKFRKLL-VPNKMQHVLCTGNLCSRETFDYLR---TL 56
E.histolytica        ----MLVLVIGDFHVPHRSAAIPQVFLDRL-NTGRIQTVLCTGNLCGKETYDILR---TL 52
S.Pombe              ----MLVLVIGDFHIPDRAPKLSEKFRQLL-IPGKISQIICLGNLTSTSVYEYLK---HV 52
S.cerevisiae         ----MLLLALSDAHIPDRATDLPVKFKKLLSVPDKISQVALLGN--STKSYDFLKFVNQI 54
                          *:* :.* ::*.*.  :.  * . *  ..::  :   **    . .: *:    :

H.sapiens            AGDVHIVRGDFDEN-LN----------------YPEQKVVTVGQFKIGLIHGHQVIPWGD 95
M.musculus           AGDVHIVRGDFDEN-LN----------------YPEQKVVTVGQFKIGLIHGHQVIPWGD 95
D.rerio              AGDVHIVRGDFDEN-LN----------------YPEQKVVTVGQFKIGLIHGHQVIPWGD 95
D.melanogaster       ANDVHIVRGDFDEN-LT----------------YPEQKVVTVGQFRIGLCHGHQVVPRGD 95
A.thaliana           CPDLHIVRGEFDED-AR----------------YPENKTLTIGQFKLGLCHGHQVIPWGD 96
C.elegans            SSDVHIVRGEFDDETLK----------------YPDTKVVTVGQFRIGVCHGHQIIPWGD 100
E.histolytica        AREVHVVKGDFDEM-QG----------------LNETEVIKIGNFKIGLMHGHQVIPWGD 95
S.Pombe              CSDLKLVKGAFDIS-SK----------------APIAGKITLGSFKIGYTNGHLVVPQDS 95
S.cerevisiae         SNNITIVRGEFDNGHLPSTKKDKASDNSRPMEEIPMNSIIRQGALKIGCCSGYTVVPKND 114
                     . :: :*:* **                           :  * :::*   *: ::* ..

H.sapiens            MASLALLQRQFDVDILISGHTHKFEAFEHENKFYINPGSATGA------YNALETN--II 147
M.musculus           MASLALLQRQFDVDILISGHTHKFEAFEHENKFYINPGSATGA------YNALETN--II 147
D.rerio              MASLALLQRQLDVDILISGHTHKFEAFENENKFYINPGSATGA------YSALESN--IT 147
D.melanogaster       PEALALIQRQLDVDILITGHTYKFEAYEHGNKFYINPGSATGA------FNPLDTN--VV 147
A.thaliana           LDSLAMLQRQLGVDILVTGHTHQFTAYKHEGGVVINPGSATGA------YSSINQD--VN 148
C.elegans            QRMLELLARQLDVDILVTGNTYECSAVEKNGRFFVDPGSATGS------FSVTKTEP-TT 153
E.histolytica        REALAIYQRQLDVDILITGHTHKLETKEVGGKYFLNPGSATGA------YSPLVDN--PV 147
S.Pombe              PEALSILAREMDADILLFGGTHKFAAYELDGCFFVNPGSATGAP----NVSAVEDDEKIV 151
S.cerevisiae         PLSLLALARQLDVDILLWGGTHNVEAYTLEGKFFVNPGSCTGA(79aa)MSDSDINGSNS 247
                        *    *::..***: * *::  :    .   ::***.**:       .    :

H.sapiens            PSFVLMDIQASTVVTYVYQLIGDDVKVERIEYKKP------- 182
M.musculus           PSFVLMDIQASTVVTYVYQLIGDDVKVERIEYKKS------- 182
D.rerio              PSFVLMDIQASTVVTYVYQLIGDDVKVERIEYKKS------- 182
D.melanogaster       PSFVLMDIQSTTVVTYVYQLIGDEVKVERIEYKKI------- 182
A.thaliana           PSFVLMDIDGFRAVVYVYELIDGEVKVDKIEFKKPPTTSSGP 190
C.elegans            PSFALLDVQADNVVTYLYRLIDDAVKVDRIIYKKSKTT---- 191
E.histolytica        PSFMLLEINDSELTIYEYTLVDGSVKCERVDFNKKQQQ---- 185
S.Pombe              PSFVLMDVQGAVLILYVYRIFDGEVRVEKMQYRKPE------ 187
S.cerevisiae         PSFCLLDIQGNTCTLYIYLYVNGEVKVDKVVYEKE------- 282
                     *** *::::      * *  ... *: ::: :.*
```

Supplementary Figure 5. Sequence alignments of TBC1d5-Ins1 and VPS29 from representative organisms.

Mutiple TBC1d5-Ins1 (A) and VPS29 (B) sequence are aligned using ClustalW (* for invariant, : for conserved, . for less conserved changes). Residues at the interface are highlited in machaccino. Residues whose mutation disrupts the binding in Figure 5 are labeled with black triangles on top.

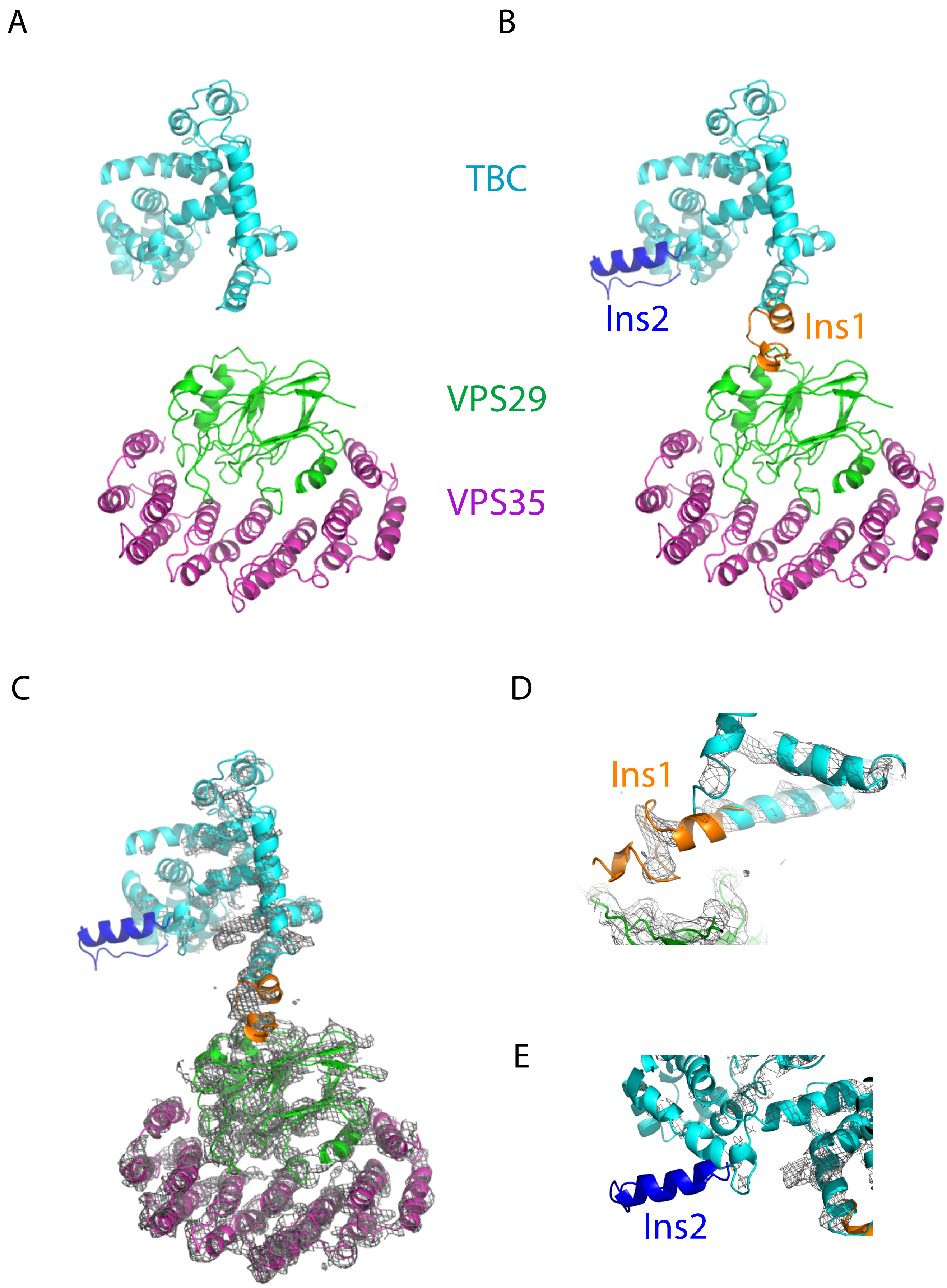
A
B
TBC
Ins2
Ins1
VPS29
VPS35
C
D
Ins1
E
Ins2

Supplementary Figure 6. Crystal structure of the ternary complex formed by TBC1d5, VPS29, and the C-terminus of VPS35.

(A) Crystal structure of the ternary complex determined at 3.8 Å. TBC represents the part that we could build with high confidence. Ins1 and Ins2 could not be confidently built in the model due to poor electron density (see panels C-E).

(B) crystal structure of TBC1d5^{TBC}/VPS29/VPS35^{C} with modeled Ins1 and Ins2. Ins1 is genrated by superimposing two VPS29 in the high resolution structure with the low resolution one Ins2 is generated in silico with PHYRE2 Protein Fold Recognition Server (http://www.sbg.bio.ic.ac.uk/phyre2/) since its structural information is lacking.

(C) Overlay between the ternary model shown in (B) with a 2Fo-Fc map at 3.8 Å resolution and contoured at 1.5 σ.

(D) Overlay between regions surrounding Ins1 of TBC1d5 with a 2Fo-Fc map at 3.8 Å resolution and contoured at 1.5 σ.

(E) Overlay between regions surrounding Ins1 of TBC1d5 with a 2Fo-Fc map at 3.8 Å resolution and contoured at 1.5 σ.

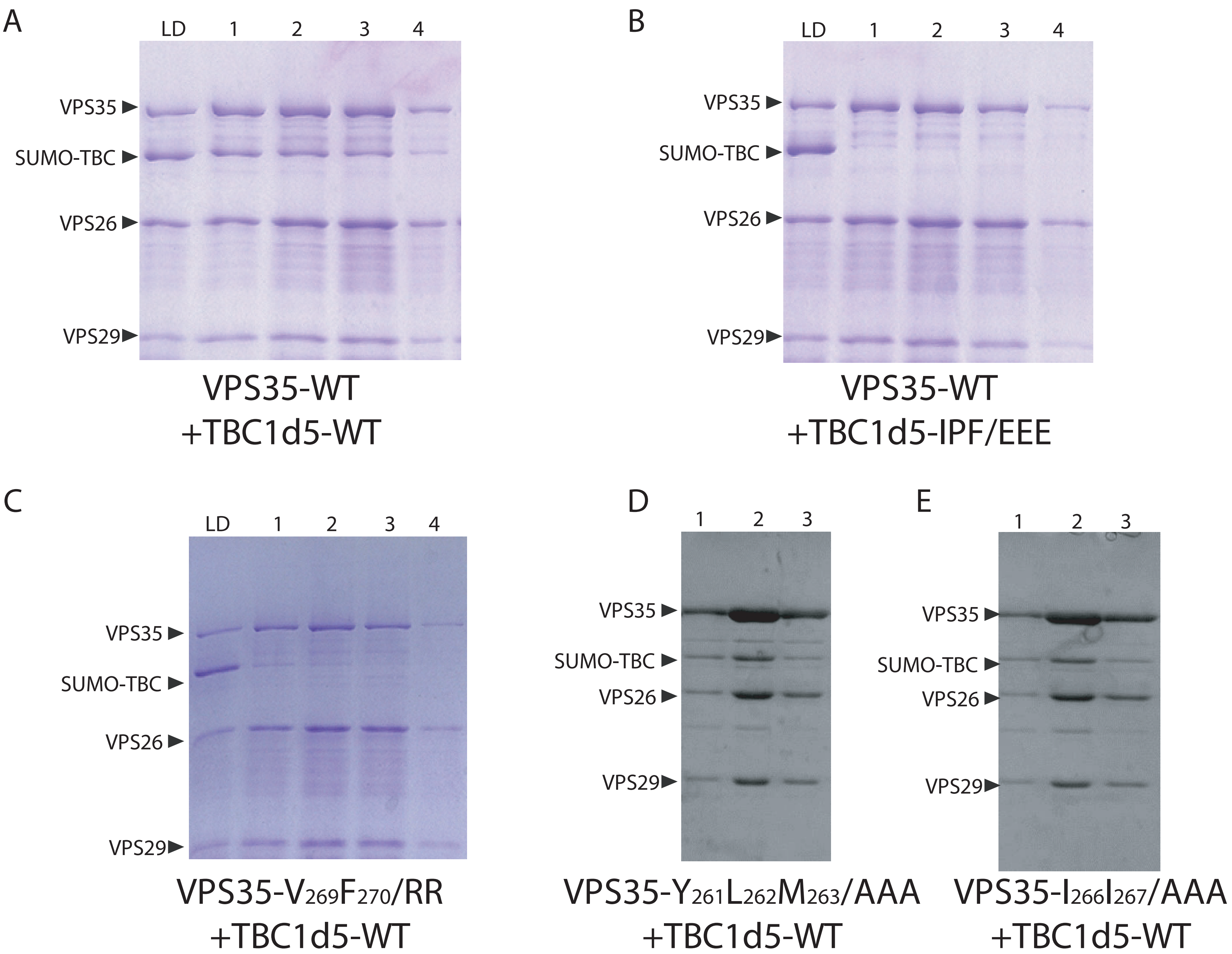
A
LD
1
2
3
4
VPS35
SUMO-TBC
VPS26
VPS29
VPS35-WT
+TBC1d5-WT
B
LD
1
2
3
4
VPS35
SUMO-TBC
VPS26
VPS29
VPS35-WT
+TBC1d5-IPF/EEE
C
LD
1
2
3
4
VPS35
SUMO-TBC
VPS26
VPS29
VPS35-V269F270/RR
+TBC1d5-WT
D
1
2
3
VPS35
SUMO-TBC
VPS26
VPS29
VPS35-Y261L262M263/AAA
+TBC1d5-WT
E
1
2
3
VPS35
SUMO-TBC
VPS26
VPS29
VPS35-I266I267/AAA
+TBC1d5-WT

Supplementary Figure 7. Mutation in critical residues from Ins2 of TBC1d5 or Repeat 6 from VPS35 abolishes the co-migration of TBC1d5 and CSC.

Purified CSC were mixed with SUMO-TBC1d5^{TBC} at a molar ratio of 1:3, and subjected to Source Q chromatography. A: VPS35-WT+TBC1d5-WT; B: VPS35-WT+TBC1d5-IPF/EEE; C: VPS35-$V_{269}F_{270}$/RR+TBC1d5-WT; D: VPS35-$Y_{261}L_{262}M_{263}$/AAA+TBC1d5-WT; E:VPS35-$I_{266}I_{267}$/AA+TBC1d5-WT.

TBC1d5^{TBC}/CSC or CSC bind to Q column more strongly than TBC1d5^{TBC}, and were eluted in later fractions. Commassie blue stained SDS-PAGE gels are shown for these later fractions with or without load samples.

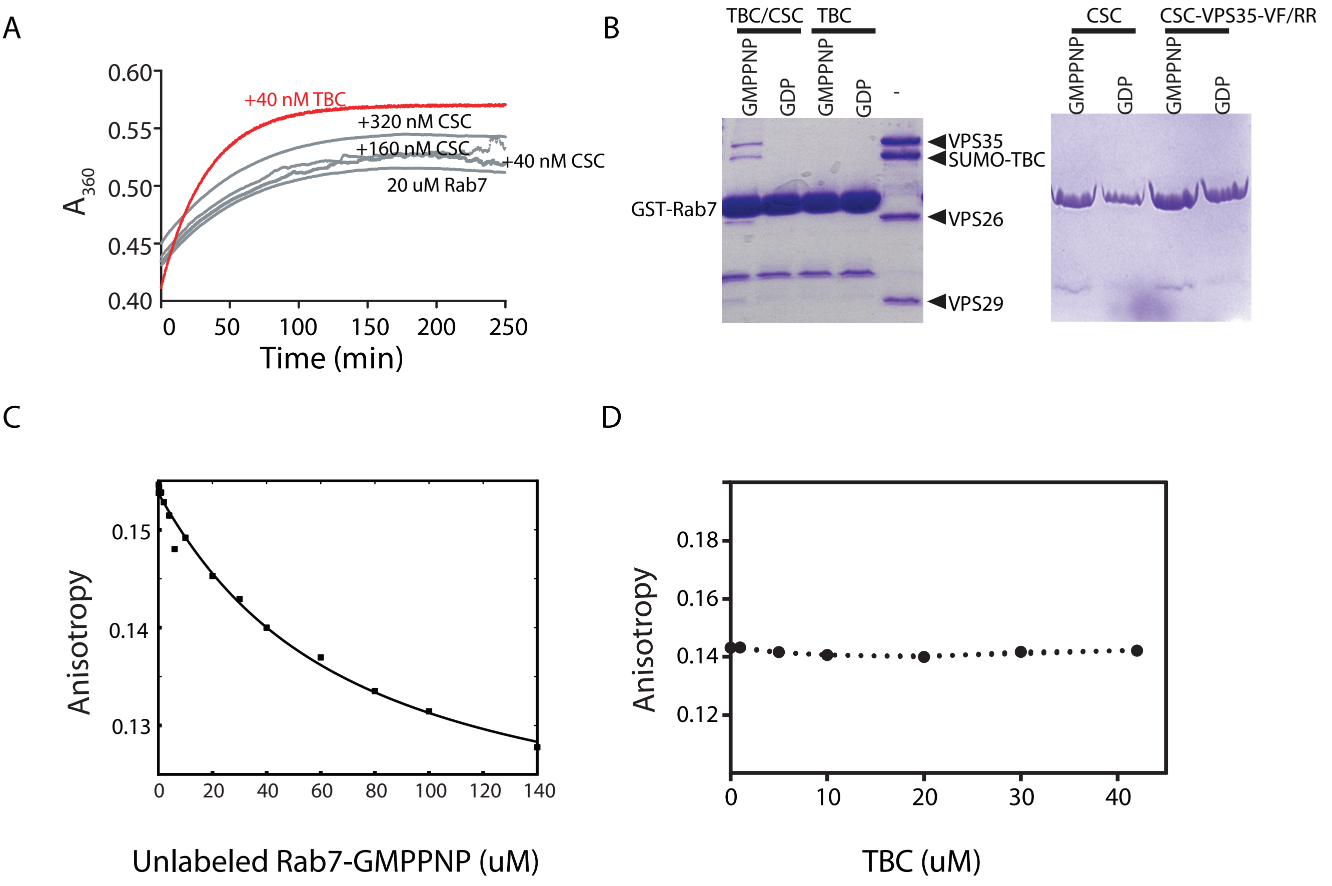

Supplementary Figure 8. Rab7 GTP hydrolysis assay and binding assay between Rab7-GMPNP and TBC1d5 TBC, CSC or TBC1d5TBC/CSC.

(A) Kinetics of GTP hydrolysis for Rab7 in the absence and presence of the TBC1d5TBC or CSC. The absorbance at 360 nm reports the amount of a conjugate of GTP hydrolysis product, Pi.

(B) GST-Rab7 pull-down of purified retromer TBC/CSC, CSC, or CSC-VPS35-VF/RR. Shown is Coomassie blue stained SDS-PAGE gels of bound samples.

(C) Fluorescence anisotropy competition binding assay, in which unlabeled Rab7-GMPPNP was added into reactions containing 8 μM TBC1d5TBC/CSC and 50 nM labeled Rab7-GMPPNP. Changes in fluorescence anisotropy (square) were used to calculate Kd using competitive binding-one site model.

(D) Fluorescence anisotropy competition binding assay, in which TBC1d5TBC was added into reactions containing 8 μM TBC1d5TBC/CSC and 50 nM labeled Rab7-GMPPNP.

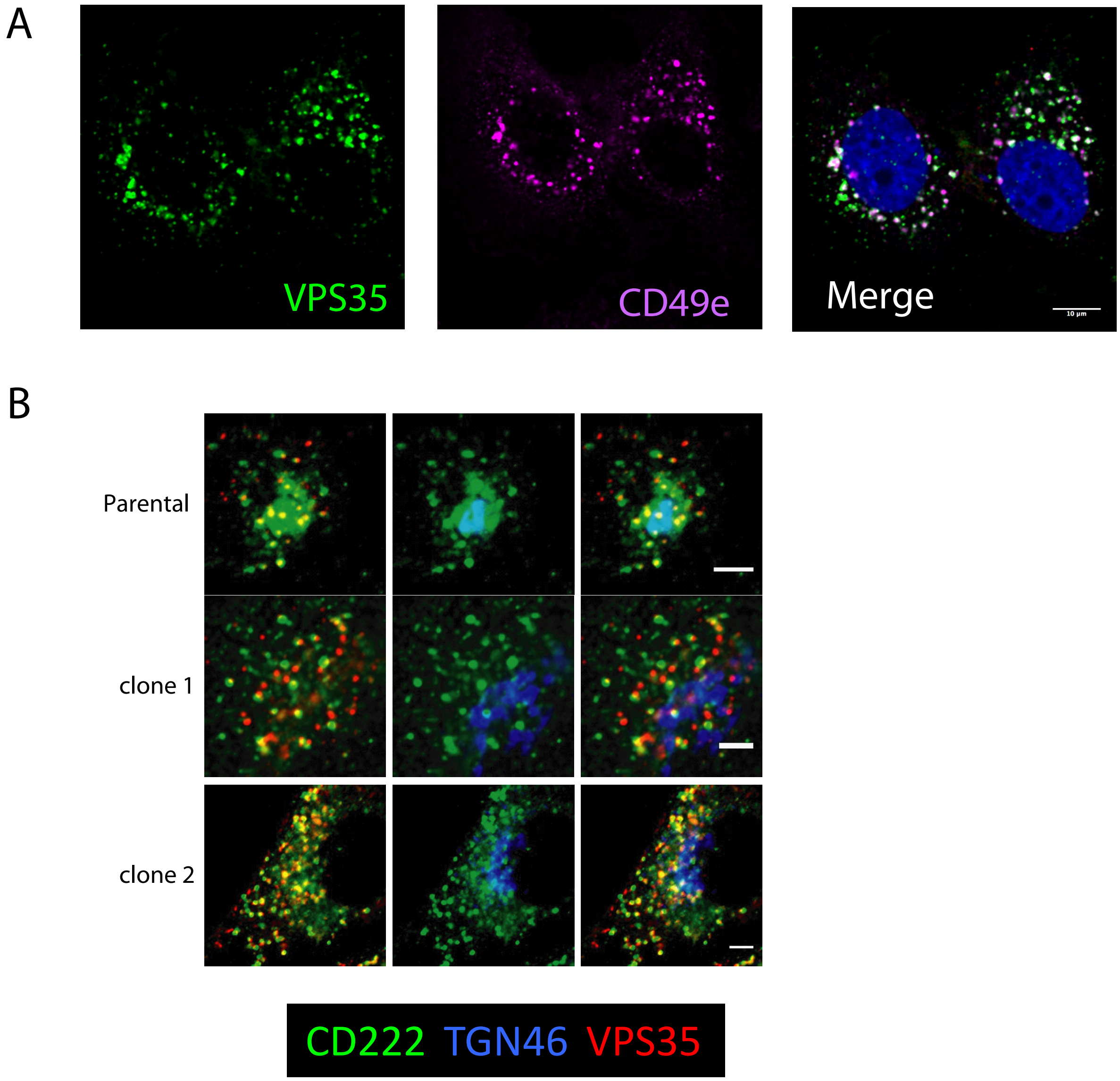

Supplementary Figure 9. TBC1d5 is required for retromer-mediated trafficking

(A) TBC1d5 knockout cells (clone 2) were incubated with anti-CD49e antibody for one hour at 37 °C. The cells were washed in 1X PBS, fixed and stained for TBC1d5 (red – not shown),VPS35 (green), and CD49e (magenta). Scale bar = 10 um

(B) Wild type HeLa cells or TBC1d5 knockout cells (clone 1 and clone 2) were grown on coverslips and stained for CD222 (CIM(6)PR) (green), TGN46 (blue) and VPS35 (red). Scale bar = 5 um

A

	VPS29	TBC1d5
H.sapiens	100	100
M.musculus	99	92
D.rerio	96	75
D.melanogaster	83	35
A.thaliana	65	27
C.elegans	59	36
E.histolytica	56	29
S.Pombe	47	20
S.cerevisiae	43	-

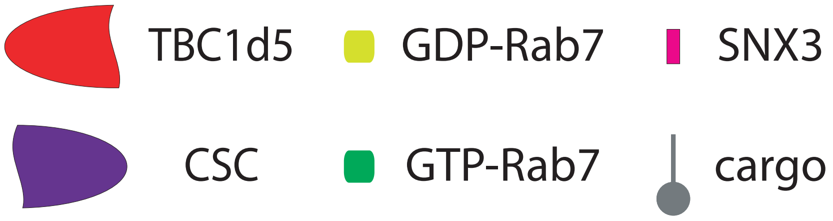

B

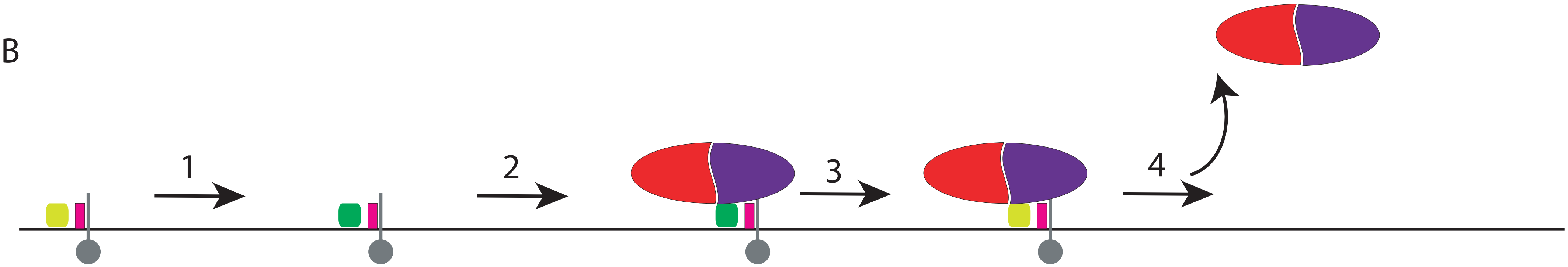

Supplementary Figure 10. Sequence conservation of VPS29 and TBC1d5, and role of Rab7 and TBC1d5 in the assembly and turnover of retromer.

(C) Sequence conservation of VPS29 and TBC1d5 among representative eukaryotic organisms. The number of sequence identity with human VPS29 and the TBC domain of TBC1d5 are listed.

(D) A proposed model for the role of Rab7 and TBC1d5 in the formation of retromer-coated tubules. SNX, WASH complex, and other known regulators are omitted for simplicity. Step 1: conversion of Rab7-GDP to Rab7-GTP; Step 2: recruitment of CSC through Rab7-GTP; Step 3: TBC1d5 is recruited via its interaction with CSC; step 4: TBC1d5 promotes GTP hydrolysis by Rab7; Step 5: GTP hydrolysis triggers the release of TBC1d5 and CSC from membrane. Although in our cartoon TBC1d5 and CSC stay together after leaving the membrane, we do not know exactly their state.

Supplementary Table 1. SAXS data Collection and Analysis.

	VPS35/VPS29	VPS35/VPS29/TBC1 d5^{TBC}
**Data q-range ($Å^{-1}$)[a]**	0.009 – 0.3	0.009 – 0.3
**Concentration (mg/ml)**	1.2	0.6
**Exposure Time (h)**		
**Sample**	12	12
**Buffer**	12	24
**R_G (Å)**	52.5(5)	50.7(5)
**D_{max} (Å)**	179	174
**MW (kDa)**	113	162
**MW (Io) (kDa) (N)**	122 (1)	182 (1)
**MW (Porod) (kDa) (N)**	155 (1)	196 (1)
**DAMMIF NSD(15) (Å)**	0.76(9)	0.98(8)
**DLS:**	Monodisperse	Monodisperse
**Apparent Rg (Å)**	53	59
**Apparent MW (kDa)**	208	260

The MW measured in the DLS is about 60% higher, probably due to the rod-like molecular shape's larger Rg relative to the assumed spherical particle.

Supplementary Table 2. DNA Constructs Used in this Study.

Construct name	Description[#1]	Source or reference
**CSC**		
VPS35	GST-Tev-VPS35-full length, GST-Tev finally removed	(Jia et al., 2012)
VPS26	His6-Tev-VPS26A (9-327), His6-Tev finally removed	(Jia et al., 2012)
VPS29	His6-Tev-VPS29-full length, His6-Tev finally removed	(Jia et al., 2012)
MBP-VPS29	MBP-TEV-VPS29	(Jia et al., 2012)
VPS35^{C}	GST-Tev-VPS35 (476-796), GST-Tev finally removed	This study
Polycistronic CSC[#2]	VPS26-VPS29-VPS35-His6	(Tabuchi et al., 2010)
VPS35_VF/RR [#2]	VPS26-VPS29-VPS35 (V269F270/RR)-His6	This study
VPS29_L2A	His6-Tev-VPS29-full length_L2A, His6-Tev finally removed	This study
VPS29_L25A	His6-Tev-VPS29-full length_L25A, His6-Tev finally removed	This study
VPS29_L152A	His6-Tev-VPS29-full length_L152A, His6-Tev finally removed	This study
VPS29_L152E	His6-Tev-VPS29-full length_L152E, His6-Tev finally removed	This study
VPS35 WT	pCMS3-H1p.shVPS35-HA-YFP-VPS35 WT suppression/re-expression	(Liu et al., 2012)
VPS35Δ6	pCMS3-H1p.shVPS35-HA-YFP-VPS35 Δ6 suppression/re-expression	(Liu et al., 2012)
**TBC**		
TBC1d5	GST-Tev-TBC1d5-full length (isoform b, 795aa)	This study
TBC1d5^{F1}	GST-Tev-TBC1d5 (1-548)	This study
TBC1d5^{TBC}	GST-Tev-TBC1d5 (1-419), GST-Tev finally removed for experiments other than GST pull-down	This study
TBC1d5^{F2}	GST-Tev-TBC1d5 (420-548)	This study
TBC1d5^{TBC}_ΔIns1	GST-Tev-TBC1d5 (1-129-(GGS)4-147-419)	This study
TBC1d5^{TBC}_ΔIns2	GST-Tev-TBC1d5 (1-263-(GGS)4-289-419)	This study
TBC1d5^{TBC}_NPL/EEE	GST-Tev-TBC1d5 (1-419)_N140P141L142/EEE	This study
TBC1d5^{TBC}_N140A	GST-Tev-TBC1d5 (1-419)_N140A	This study
TBC1d5^{TBC}_P141A	GST-Tev-TBC1d5 (1-419)_P141A	This study
TBC1d5^{TBC}_L142A	GST-Tev-TBC1d5 (1-419)_L142A	This study
TBC1d5^{TBC}_L142E	GST-Tev-TBC1d5 (1-419)_L142E	This study
TBC1d5^{TBC}_L142W	GST-Tev-TBC1d5 (1-419)_L142W	This study
TBC1d5^{TBC}_W150A	GST-Tev-TBC1d5 (1-419)_W150A	This study
Ins1	MBP-Tev-TBC1d5 (132-158)-His6, MBP-Tev finally removed	This study
GST-Ins1	GST-Tev-TBC1d5 (132-158)	This study
SUMO-TBC	His6-SUMO-TBC1d5 (1-419)	This study
SUMO-TBC-IPF/EEE	His6-SUMO-TBC1d5 (1-419)-I283P284F285/EEE	This study
shRNA	5' – GAAGCCATATCGCAGAGCTA – 3'<br>Sequence used in pCMS3-H1p.shTBC1d5-HA-YFP vector	This study
TBC1d5 WT	pCMS3-H1p.shTBC1d5-HA-YFP-TBC1d5 WT suppression/re-expression	This study
TBC1d5 ΔIns1	pCMS3-H1p.shTBC1d5-HA-YFP-TBC1d5 ΔIns1 suppression/re-expression	This study
TBC1d5 ΔIns2	pCMS3-H1p.shTBC1d5-HA-YFP-TBC1d5 ΔIns2 suppression/re-expression	This study
TBC1d5 ΔIns1+2	pCMS3-H1p.shTBC1d5-HA-YFP-TBC1d5 ΔIns1+2 suppression/re-expression	This study

TBC1d5 NPL/EEE	pCMS3-H1p.shTBC1d5-HA-YFP-TBC1d5 NPL/EEE suppression/re-expression	This study
TBC1d5 L142E	pCMS3-H1p.shTBC1d5-HA-YFP-TBC1d5 L142E suppression/re-expression	This study
TBC1d5 W150A	pCMS3-H1p.shTBC1d5-HA-YFP-TBC1d5 W150A suppression/re-expression	This study
shTBC1d5	pCMS3-H1p.shTBC1d5-HA-YFP suppression/re-expression	This study
TBC1d5^{TBC} WT	pCi2.Flag-YFP- TBC1d5^{TBC} WT expression vector	This study
TBC1d5^{TBC} LR/AA	pCi2.Flag-YFP- TBC1d5^{TBC} LR/AA expression vector	This study
TBC1d5^{TBC} L142A	pCi2.Flag-YFP- TBC1d5^{TBC} L142A expression vector	This study
TBC1d5^{TBC} IPF/EEE	pCi2.Flag-YFP- TBC1d5^{TBC} IPF/EEE expression vector	This study
**Rab**		
Rab7 WT	GST-Tev-Rab7 (1-186), GST-Tev finally removed for experiment other than GST pull-down	This study
Rab7[#3]	GST-Tev-Rab7 (1-186-Cys and Cys143Ser), GST-Tev finally removed	This study
**Other**		
FAM21	MBP-Tev-FAM21 (1278-1341)	(Jia et al., 2012)
SNX3	MBP-Tev-SNX3-full length	This study

Note:

#1 All cDNAs are from human, and sequences were confirmed by DNA sequencing.

#2 Used for co-purification with SUMO-TBC.

#3 Labeled with Alexa Fluor 488 and used for fluorescence anisotrpy experiments.

Supplementary Table 3. Summary of Antibodies Used in this Study

Antibody	Company	Cat #
TBC1d5	Proteintech Group	17078-1-AP
VPS35	Abcam	ab10099-100
GFP	Invitrogen	A11120
VPS26	Epitomics	S1181
VPS29	GeneTex	GTX104768
Actin	Santa Cruz	SC1616
CD49e	BD	555615
CD222	Serotec	MCA2048T

References

Fischer, H., Neto, M.D., Napolitano, H.B., Polikarpov, I., and Craievich, A.F. (2010). Determination of the molecular weight of proteins in solution from a single small-angle X-ray scattering measurement on a relative scale. Journal of Applied Crystallography *43*, 101-109.

Gomez, T.S., and Billadeau, D.D. (2009). A FAM21-containing WASH complex regulates retromer-dependent sorting. Dev Cell *17*, 699-711.

Hansen, S. (2012). BayesApp: a web site for indirect transformation of small-angle scattering data. Journal of Applied Crystallography *45*, 566-567.

Jia, D., Gomez, T.S., Billadeau, D.D., and Rosen, M.K. (2012). Multiple repeat elements within the FAM21 tail link the WASH actin regulatory complex to the retromer. Mol Biol Cell *23*, 2352-2361.

Konarev, P.V., Volkov, V.V., Sokolova, A.V., Koch, M.H.J., and Svergun, D.I. (2003). PRIMUS: a Windows PC-based system for small-angle scattering data analysis. Journal of Applied Crystallography *36*, 1277-1282.

Kozin, M.B., and Svergun, D.I. (2001). Automated matching of high- and low-resolution structural models. Journal of Applied Crystallography *34*, 33-41.

Liu, T.T., Gomez, T.S., Sackey, B.K., Billadeau, D.D., and Burd, C.G. (2012). Rab GTPase regulation of retromer-mediated cargo export during endosome maturation. Mol Biol Cell *23*, 2505-2515.

Rambo, R.P., and Tainer, J.A. (2013). Accurate assessment of mass, models and resolution by small-angle scattering. Nature *496*, 477-481.

Tabuchi, M., Yanatori, I., Kawai, Y., and Kishi, F. (2010). Retromer-mediated direct sorting is required for proper endosomal recycling of the mammalian iron transporter DMT1. J Cell Sci *123*, 756-766.

Volkov, V.V., and Svergun, D.I. (2003). Uniqueness of ab initio shape determination in small-angle scattering. Journal of Applied Crystallography *36*, 860-864.
